# Melatonin-Associated Facial Swelling in an Oncology Patient: Case Report and Review of Swelling of the Face in Individuals With Head and Neck Cancer

**DOI:** 10.7759/cureus.10866

**Published:** 2020-10-09

**Authors:** Ravi C Patel, Shelby L Kubicki, Philip R Cohen, Deborah F MacFarlane

**Affiliations:** 1 Dermatology, MD Anderson Cancer Center, Houston, USA; 2 Dermatology, University of Texas McGovern Medical School, Houston, USA; 3 Dermatology, San Diego Family Dermatology, National City, USA

**Keywords:** melatonin, lymphedema, facial swelling, angioedema

## Abstract

Facial swelling has several etiologies. In patients with head and neck malignancies, this can include primary disease progression or iatrogenic causes. A 66-year-old man presented with increased facial swelling and erythema for 18 months. He had a history of baseline postoperative facial lymphedema following head and neck surgery and radiotherapy for desmoplastic melanoma approximately 20 years ago. However, his facial edema acutely worsened 18 months prior to presentation. A medication review revealed that he was regularly taking melatonin for the past two years. Approximately two weeks after cessation of melatonin therapy, the patient’s facial appearance returned to baseline. In conclusion, it is important for clinicians to perform a thorough medication review for patients with facial swelling and erythema.

## Introduction

Facial edema is frequently encountered in patients treated for cancer of the head and neck region. There are several potential causes. They include cancer-related reasons, prior management of the malignancy, and systemic therapies.

We present a man with a history of bilateral neck dissection and postoperative facial lymphedema following surgery and radiotherapy for desmoplastic melanoma of his lower lip who was noted to have developed increased facial swelling and erythema. A review of possible etiologies revealed a history of melatonin intake prior to the onset of his symptoms. Discontinuation of this medication resulted in the resolution of the newly increased facial edema.

Melatonin, albeit uncommon, should be considered in the differential diagnosis of facial edema in oncology patients.

## Case presentation

**Figure 1:**
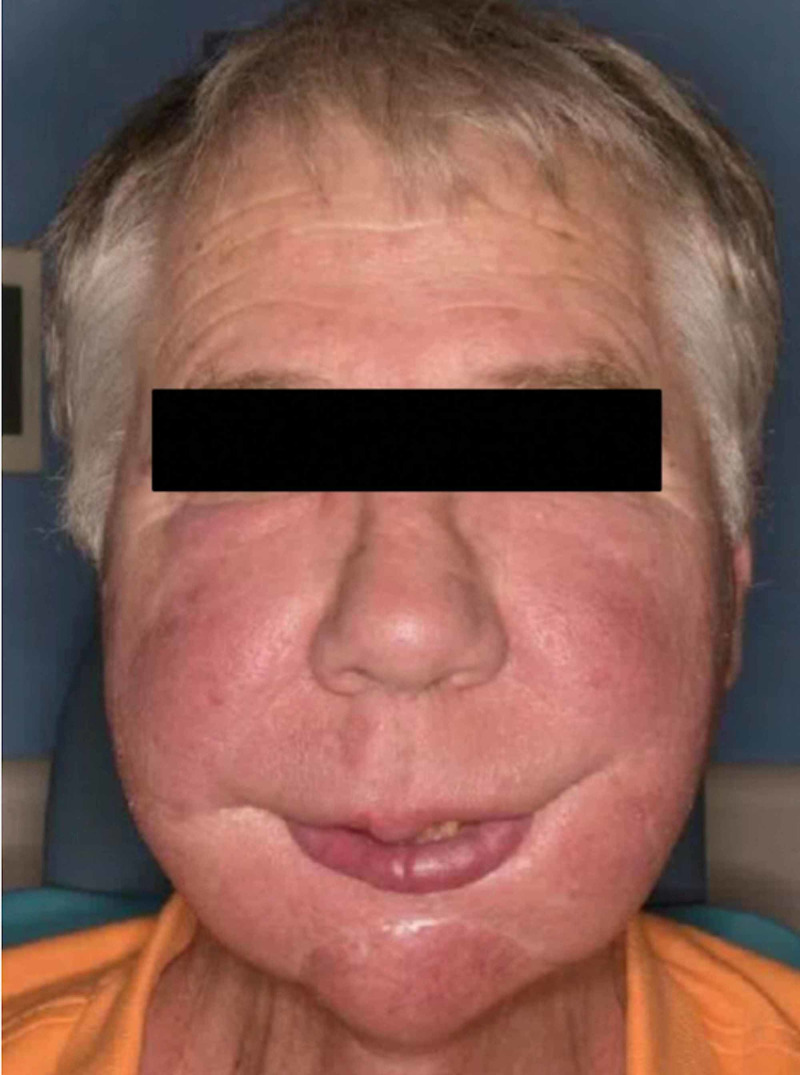
Melatonin-associated facial swelling and erythema

A 66-year-old man presented with increased facial swelling and erythema (Figure 1). He had baseline postoperative facial lymphedema following resection of a desmoplastic melanoma of the lower lip, lymph node dissection, and radiation approximately 20 years ago. The facial edema acutely worsened 18 months prior to his presentation. He reported exacerbation by heat and no improvement with cetirizine.

**Figure 2:**
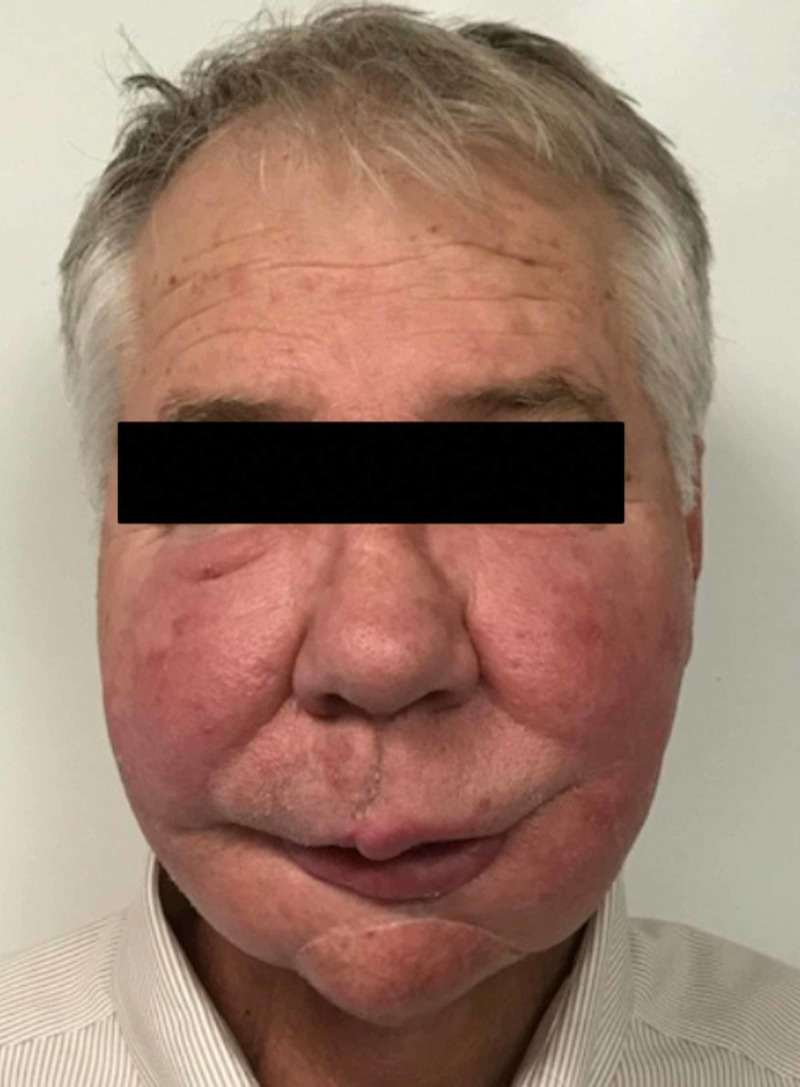
Facial appearance returned to baseline following cessation of melatonin

Review of his medications revealed initiation of melatonin two years earlier for insomnia. He was not taking any other supplements. Discontinuation was advised; the excess swelling and erythema resolved two weeks after cessation. At four-month follow-up, his appearance had returned to baseline (Figure 2).

## Discussion

Oncology patients with head and neck malignancies can develop facial edema. Postoperative lymphedema can present with swelling and erythema. The associated lymphatic stasis also can promote bacterial growth and an increased risk for cellulitis. Luebbers et al. described a 45-year-old man with demodicidosis that presented with sudden onset facial swelling and erythema six months after resection of a squamous cell carcinoma, bilateral neck dissection, and chemoradiotherapy [1].

Venous outflow obstruction secondary to tumor metastasis in cancer patients may cause facial swelling. Superior vena cava syndrome presents clinically with facial edema, shortness of breath, and headache [2]. While intrathoracic tumors are the most common causes of superior vena cava syndrome, metastatic melanoma has been shown to obstruct the superior vena cava and produce these symptoms [2-4].

In a surgical setting, placement of an emergency airway has occasionally been required following bilateral neck dissection. Extubation can provoke facial and laryngeal edema. Laryngeal edema and subsequent obstruction are not limited to the immediate postoperative period; it has been reported up to ten years after neck dissection in a 75-year-old woman following resection of a squamous cell carcinoma of the tongue [5].

A review of the patient’s medication is an important initial step when assessing the new onset of facial swelling and erythema. Multiple drug classes can produce angioedema. Drugs commonly associated with angioedema include angiotensin-converting enzyme inhibitors, beta-lactam antibiotics, and nonsteroidal anti-inflammatory drugs [6].

Melatonin is secreted endogenously by the pineal gland and regulates the circadian rhythm [7]. Individuals electively use melatonin for the management of primary sleep disorders. The most commonly reported melatonin-induced adverse events include dizziness, drowsiness, headache, and nausea [7].

Suhner et al. reported extremity swelling and flushes with the use of melatonin [8]. Symptoms of angioedema such as difficulty breathing and swallowing have also been reported [9]. In 2006, the Food and Drug Administration (FDA) requested for all drugs approved for the treatment of sleep disorders to modify their labels to include warnings about anaphylaxis and angioedema [10].

The mechanism of melatonin-induced facial swelling remains to be determined. However, drug-induced angioedema associated with facial swelling has the potential to progress to laryngeal edema and airway obstruction. Therefore, patients experiencing these symptoms should discontinue melatonin therapy.

## Conclusions

Facial edema in head and neck cancer patients can result from not only their neoplasm and its management but also from ancillary medications. Our patient’s initiation of melatonin to treat insomnia resulted in increased facial swelling and erythema which subsequently resolved once he discontinued the drug. Therefore, the new onset of facial swelling and erythema should prompt the clinician to consider the possibility of a drug-associated etiology.
